# Matching Occupational Injury and Illness Data Using Augmented Twin Neural Networks

**DOI:** 10.23889/ijpds.v9i5.2868

**Published:** 2024-09-18

**Authors:** Elan Segarra

**Affiliations:** 1 U.S. Bureau of Labor Statistics

When record linkage efforts involve complex characteristics there is ample potential for general purpose machine learning (ML) techniques to succeed where traditional probabilistic approaches might fall short. However, there can still be pre-processing (e.g. geocoding) and hand-picked comparators that can further improve linkage outcomes using standard ML models. In this project we present a fusion of these sides we are calling an Augmented Twin Neural Network. This approach leverages the inherent flexibility of Twin Neural Networks in a record linkage context while adding additional layers to allow for hand curated comparators that may be difficult for ML optimizers to implicitly identify without sufficiently large, labeled data sets. The framework is used to match establishments from the BLS Survey of Occupational Injuries and Illnesses to establishments in the OSHA Injury Tracking Application data. The difficulties inherent in matching company names and addresses and the existence of multi-establishment firms make this a prime application for testing. Linkage outcome metrics of this augmented algorithm are compared both with results from probabilistic methods (e.g. Fellegi-Sunter) and standard machine learning methods to illustrate the added benefits.

